# Impact of Diabetes Mellitus on Outcomes in Patients with Left Ventricular Assist Devices

**DOI:** 10.3390/biomedicines12071604

**Published:** 2024-07-18

**Authors:** William Crugnola, Andrew Cinquina, Daniel Mattimore, Savannah Bitzas, Jonathon Schwartz, Saleem Zaidi, Sergio D. Bergese

**Affiliations:** 1 Department of Anesthesiology, Stony Brook University Hospital, Stony Brook, NY 11794, USA; william.crugnola@stonybrookmedicine.edu (W.C.); andrew.cinquina@stonybrookmedicine.edu (A.C.); daniel.mattimore@stonybrookmedicine.edu (D.M.); jonathon.schwartz@stonybrookmedicine.edu (J.S.); saleem.zaidi@stonybrookmedicine.edu (S.Z.); 2 School of Medicine, Stony Brook University, Stony Brook, NY 11794, USA; savannah.bitzas@stonybrookmedicine.edu

**Keywords:** left ventricular assist device, diabetes mellitus, chronic kidney disease, heart failure

## Abstract

Heart failure (HF) represents a significant health burden in the United States, resulting in substantial mortality and healthcare costs. Through the array of treatment options available, including lifestyle modifications, medications, and implantable devices, HF management has evolved. Left ventricular assist devices (LVADs) have emerged as a crucial intervention, particularly in patients with advanced HF. However, the prevalence of comorbidities such as diabetes mellitus (DM) complicates treatment outcomes. By elucidating the impact of DM on LVAD outcomes, this review aims to inform clinical practice and enhance patient care strategies for individuals undergoing LVAD therapy. Patients with DM have higher rates of hypertension, dyslipidemia, peripheral vascular disease, and renal dysfunction, posing challenges to LVAD management. The macro/microvascular changes that occur in DM can lead to cardiomyopathy and HF. Glycemic control post LVAD implantation is a critical factor affecting patient outcomes. The recent literature has shown significant decreases in hemoglobin A1c following LVAD implantation, representing a possible bidirectional relationship between DM and LVADs; however, the clinical significance of this decrease is unclear. Furthermore, while some studies show increased short- and long-term mortality in patients with DM after LVAD implantation, there still is no literature consensus regarding either mortality or major adverse outcomes in DM patients.

## 1. Introduction

The prevalence of heart failure (HF) in the United States has been estimated at 6 million people between 2015 and 2018 [1]. There were 83,616 deaths attributed to HF in 2018 [1]. Additionally, the lifetime risk for HF is estimated at 20–45% from ages 45 to 95 years old [2]. This high disease prevalence is also associated with a growing medical cost, with 200,000 more hospitalizations due to HF in 2018 compared to 2008 in the United States alone [3]. Including indirect costs, hospitalizations due to HF were estimated at USD 30.7 billion in 2012 and are expected to rise in the United States to USD 69.8 billion by 2030 [4].

HF is caused by structural or functional impairment of the ventricular filling or ejection of blood, leading to the inability of the heart to meet the body’s need for blood and oxygen [5]. Multiple treatment options exist for HF depending on the severity, including lifestyle changes, medication management, or implantable devices (i.e., biventricular pacemaker or implantable cardioverter-defibrillator (ICD) ± cardiac resynchronization therapy). Temporary assistance can be provided via mechanical cardiac support devices such as the Impella^®^, intra-aortic balloon pump, or extracorporeal membrane oxygenation (ECMO), often acting as the last resort for cardiopulmonary support in critically ill patients [6]. In HF patients with persistent, irreversible hypoperfusion/congestion despite optimal medical therapy, the long-term treatment options are left ventricular assist devices (LVADs) and cardiac transplantation. Patients who have reached this point often require continuous ionotropic support [7].

Approximately 40% of patients with HF also have diabetes mellitus (DM), representing a significant comorbidity for these patients [8]. A study of patients who underwent LVAD placement between 2011 and 2014 found that 34% had DM [9]. Additionally, the prevalence of peripheral vascular disease, hypertension (HTN), renal failure, ischemic cardiomyopathy, and dyslipidemia was significantly greater in patients with DM vs. non-diabetic patients [9].

## 2. Diabetes Mellitus and Heart Failure

Type 1 DM (T1DM) is the less common form of DM, accounting for about 5–10% of diabetic cases [10]. T1DM is an autoimmune disorder characterized by the T-cell-mediated destruction of pancreatic β-cells, influenced by genetic and environmental factors [11]. Glutamic acid decarboxylase autoantibodies, markers of this disease, destroy pancreatic β-cells, resulting in insulin deficiency and subsequent uncontrolled hyperglycemia [12]. T1DM is often diagnosed in childhood, but late onset can occur in adults. Type 2 DM (T2DM) is the most common form of DM, influenced by obesity and unhealthy lifestyles. T2DM has two major components: insulin resistance and pancreatic β-cell dysfunction [13]. The overproduction of insulin by β-cells compensates for the decreased insulin sensitivity in peripheral tissues [14]. However, the β-cells eventually become dysfunctional, leading to insufficient production, reduced levels of circulating insulin, and uncontrolled hyperglycemia [10].

DM is a progressive disorder that can lead to long-term micro- and macrovascular complications. DM is a well-established cardiovascular disease (CVD) risk factor for conditions such as coronary artery disease (CAD), peripheral artery disease, stroke, HF, retinopathy, nephropathy, and cardiac autonomic neuropathy. Adults with DM have two to four times increased cardiovascular risk compared to those without DM, and the risk is directly correlated with worsening glycemic control [15]. In patients with DM, CVD is the leading cause of morbidity and mortality, with myocardial infarction as the leading cause of death [16,17]. These macrovascular complications are due to the accelerated atherosclerotic process in DM as glucose control worsens. DM patients have over 20% risk per 10 years for major coronary events, placing these individuals in the highest risk category. This transition to a high-risk coronary event category occurs 15 years earlier for men and women with DM compared to non-diabetics [18].

**Figure 1:**
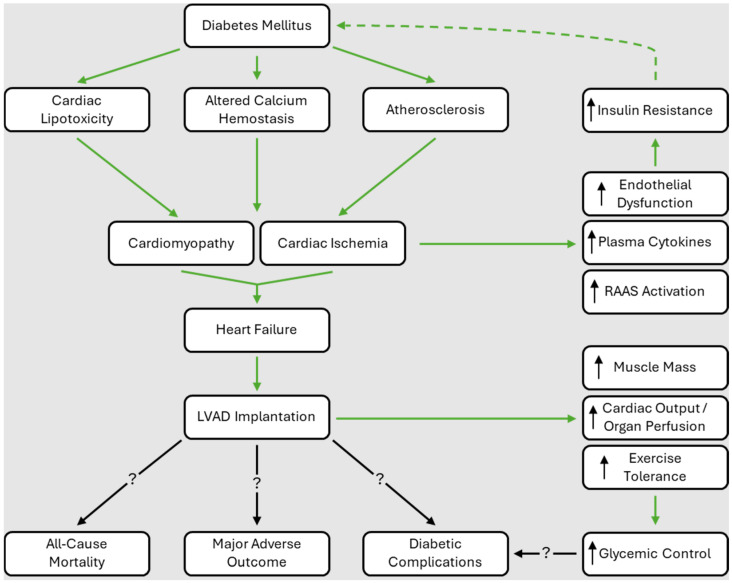
Summary of the relationship between diabetes mellitus (DM), diabetic complications, heart failure (HF), inflammation, glycemic control, and major outcomes/mortality after left ventricular assist device (LVAD) implantation. DM can cause hyperglycemia, leading to cardiac lipotoxicity, the disruption of calcium hemostasis, and atherosclerotic plaques. These changes can cause cardiomyopathy and/or ischemia, leading to HF. Also, HF has been associated with chronic inflammatory changes including endothelial dysfunction, cytokine production, and renin-angiotensin-aldosterone system (RAAS) activation, which can further worsen insulin resistance in a positive feedback loop (indicated by the dashed line). Severe HF may necessitate LVAD implantation. The literature has shown an improvement in glycemic control (as decreased A1c) after LVAD implantation, with increased muscle mass, improved cardiac output and organ perfusion, and/or improved exercise tolerance as potential explanations; however, the long-term clinical impact on diabetic co-morbidities remains unknown (indicated by the question mark). Additionally, there still is no literature consensus (indicated by the black lines with question marks) regarding the impact of DM on all-cause mortality and major adverse outcomes (pump thrombosis, stroke, infection, etc.) after LVAD implantation (question marks in this figure).

The macrovascular/microvascular dysfunction and metabolic derangements of DM can contribute to the development of cardiomyopathy. DM alters the myocardium metabolically, structurally, and functionally, resulting in DM-induced cardiomyopathy (DMCMP). DM decreases diastolic function due to increased cardiac triglyceride accumulation, which causes lipotoxicity and altered calcium hemostasis of the myocardium [19,20]. DMCMP can result in LV dysfunction, such as LV fibrosis and increased LV stiffness. These effects can lead to symptomatic HF, presenting as heart failure with reduced ejection fraction (HFrEF) or heart failure with preserved ejection fraction (HFpEF) [18,21]. One study found that the likelihood of having an LV mass greater than the 75th percentile is greater in patients with DM than in non-diabetics [22]. In addition to increasing the risk of developing HF, DM comorbid with HFrEF is associated with a greater hospitalization rate due to conditions such as decompensated HF, cardiovascular events, and infections compared to HFrEF patients without DM [23]. Similarly, patients with chronic HF have an increased risk of developing insulin resistance as compared to patients without HF [24]. Several proposed mechanisms for this observation include increased circulating plasma cytokines, chronic activation of the renin–angiotensin–aldosterone system (RAAS), and endothelial dysfunction [25]. This interplay between the pathology of DM and HF is further illustrated in Figure 1.

Additionally, DM is a risk factor for surgical complications, such as infection, poor wound healing, and pneumonia [26], creating an important consideration before LVAD placement. To help optimize LVAD patients pre-, peri-, and post-operatively, the International Society for Heart and Lung Transplantation developed a task force to provide guidelines for mechanical cardiac support (MCS) placement. Regarding DM, their recommendation is for all patients to be screened with fasting glucose before intervention. If the patient’s glucose is abnormal or they have known DM, an A1c level should be obtained to evaluate their glycemic control. Furthermore, they should undergo evaluation for signs of DM-related end-organ damage (i.e., peripheral neuropathy, nephropathy, proliferative retinopathy, vascular disease, etc.) as these signs of overall poor glycemic control are relative contraindications for the placement of MCS. Patients with poorly controlled DM should speak with an endocrinologist before device placement to aid in pre-op optimization [27,28,29].

Additionally, hyperglycemia in poorly controlled DM causes microvascular renal changes such as glomerular hyperfiltration, glomerular and tubulointerstitial inflammation, and changes in the extracellular matrix, leading to chronic kidney disease (CKD) [30]. Progression from CKD to end-stage renal disease (ESRD) requiring dialysis can cause the normalization of hemoglobin A1c in a phenomenon known as “burnt-out diabetes” [31]. Many factors, including decreased renal/hepatic insulin clearance, decreased renal gluconeogenesis, and malnutrition/protein wasting, contribute to this phenomenon and can place patients at increased risk of hypoglycemic events [31]. Similar mechanisms increasing the risk for hypoglycemia occur with CKD, even without ESRD. This is of particular importance to potential LVAD patients, as post-operative hypoglycemia increases the risk of morbidity and mortality after cardiac surgery [32]. Considering the unique relationship between DM and HF, we examined the existing literature to assess how pre-existing DM affects LVAD outcomes, specifically glycemic control, adverse effects, and mortality.

## 3. LVAD

### 3.1. LVAD Indications

While cardiac transplantation is a treatment option for candidate patients with advanced HF refractory to medical therapy, this option is limited by organ availability, comorbidities affecting post-transplant survival, and long waitlists [33]. Mechanical circulatory support with durable LVADs was developed as a bridge to curative heart transplantation (bridge to transplantation, BTT) or for long-term destination therapy (DT) in patients with end-stage HF. Specific indications for LVAD placement include the presence of NHYA HF class IV for at least 60–90 days, an INTERMACS profile of 7 or lower, maximally tolerated medical therapy (pharmacologic, respiratory support, and ICD placement if indicated), chronic ionotropic medication reliance, an LV ejection fraction < 25%, a systolic blood pressure ≤ 80–90 mmHg, a cardiac index ≤ 2 L/min/m^2^, worsening RV or kidney function, or a pulmonary capillary wedge pressure ≥ 20 mmHg [34,35,36]. In BTT cases, LVADs provide hemodynamic support and medical optimization for patients awaiting cardiac transplantation. In patients who are ineligible for a cardiac transplant, LVADs can serve as a DT, especially with ongoing technological advancements that allow for the increased long-term survival of HF patients [37]. Additionally, the term “bridge to decision” describes situations in which decision points are yet to be reached regarding treatment eligibility or the clarification of patient goals of care. For example, end-organ damage may initially render a patient ineligible for transplant, but eligibility may return following hemodynamic optimization from LVAD placement [37]. LVADs can also provide a bridge to recovery in the form of LVAD explantation and even the significant recovery of cardiopulmonary function beyond the preoperative HF state. Jakovljevic et al. found that 38% of explanted LVAD patients in their cohort achieved similar cardiac power output and functional capacity to healthy controls during exercise testing [38].

### 3.2. LVAD Implantation

LVAD implantation is most often performed with the heart beating and on cardiopulmonary bypass. However, it can also be performed with an aortic cross-clamp, cardioplegia, a non-beating heart, and even off bypass [39,40]. Intraoperative transesophageal echocardiography aids in evaluating RV function and significant aortic regurgitation. It can help to rule out the presence of an LV thrombus or patent foramen ovale [41]. After achieving proper systemic anticoagulation (AC), the LV apex is cored, and the inflow graft is sutured to the apex. Afterward, the pump is attached to the inflow ring. A partial occlusion clamp is then applied to the aorta, and an aortotomy is performed, allowing the outflow graft to be sutured onto the ascending aorta. Lastly, the drive line is tunneled to the mid-clavicular line, just below the costal margin [42]. The system supports the failing LV by pumping blood from the left ventricle via an apical inflow cannula to the aorta via an outflow cannula [43]. LVADs have been shown to improve survival and quality of life among patients with advanced HF, with outcomes equal or superior to those reported for cardiac transplants [43,44]. Additionally, LVADs may be underutilized, as only 2517 LVADs were implanted in 2022, despite there being an estimated 40,000 to more than 200,000 patients who may qualify for LVAD therapy [44,45].

### 3.3. LVAD Generations

LVADs are commonly subdivided into different generations which produce forward flow through various mechanisms. First-generation LVADs were initially based on pulsatile flow using pneumatic compression, but have since been replaced by continuous-flow LVAD (CF-LVAD) systems due to their improved durability, miniaturization, and survival [43]. Second-generation LVADs, such as the HeartMate II™, used axial propellers to generate continuous flow [46]. Axial-flow devices were the predominant LVAD-type implanted between 2012 and 2017 [47]. Third-generation LVADs, such as HeartWare^®^ and HeartMate III™, use centrifugal mechanisms via rotating impellers within the device to produce continuous flow via frictionless rotation, limiting stress on the blood components and associated hemolysis [46]. Whereas the impeller for HeartWare^®^ is suspended by a combination of magnetic and hydrodynamic forces, the HeartMate III™ is fully suspended using magnetic forces [46]. The hybrid magnetic/hydrodynamic device comprised 48.9% of all implants in 2018, but was subsequently taken off the market due to increased adverse neurological effects [47]. The centrifugal, fully mechanically levitated HeartMate III™ LVAD is the only type currently implanted [47].

### 3.4. LVAD Contraindications

Absolute and strong relative contraindications for the implantation of LVADs exist. Interestingly, while the reviewed literature did not list DM as an absolute contraindication to LVAD placement, guidelines do exist that will be discussed below, listing poorly controlled DM as a relative LVAD contraindication. While LV dysfunction is the most common cause of HF, patients with primary or predominant RV dysfunction will not achieve similar benefits and thus are poor LVAD implantation candidates [48]. Inadequate RV function can cause insufficient left heart filling and flow to the LVAD, increasing the risk of device malfunction/failure. Aortic regurgitation can limit the benefits of CF-LVAD placement. Without the pulsatility of normal preload and afterload with continuous flow, the aortic valve cusps can become fixed in place, facilitating increased regurgitation. Repair or replacement of the faulty valve should be considered before LVAD placement [49]. Active bleeding or a platelet count of less than 50,000 are contraindications due to an elevated perioperative bleeding risk [36]. The inability to initiate or tolerate AC after LVAD placement is also a contraindication, as the devices themselves stimulate coagulation and thrombus formation [36]. Patients with neurological dysfunction from cardiogenic shock, resulting in a compromise of “higher brain functions” (i.e., cognition, memory, behavior), may have increased survival after implantation but at the expense of increased suffering and decreased quality of life [36]. Implantation is also contraindicated if there is the presence of at least one other comorbid end-organ dysfunction (i.e., liver failure, renal failure, etc.) [36].

There are other factors that, while not absolute contraindications, should be considered before LVAD implantation. According to the 2013 International Society for Heart and Lung Transplantation, DM is a relative contraindication in the setting of poor glycemic control or related complications, such as retinopathy, vasculopathy, nephropathy, or neuropathy, although this recommendation is Class IIb with a level of evidence of C [50]. In patients with recurrent tachyarrhythmias, such as ventricular tachycardia or ventricular fibrillation, LVAD implantation can alter the heart’s electrical conduction system through direct tissue damage or post-surgical changes. These alterations could induce/worsen these arrhythmias, especially in patients predisposed to them [48]. Furthermore, screening for conditions causing or predisposing a patient to have gastrointestinal bleeding is advised, given the need for life-long AC after device implantation [48]. Anatomic variations, such as hypertrophic cardiomyopathy or a large ventricular septal defect, can compromise the position and function of the LVAD [36]. Lastly, the psychosocial characteristics of the patient should be considered. LVAD maintenance is complex and requires a requisite level of understanding and commitment to medication and device management, the presence of a caregiver if needed, and the ability to maintain adequate outpatient follow-up [36].

### 3.5. LVAD Complications

Several complications are commonly associated with LVAD placement, both peri- and post-operatively. The most common is bleeding, which can be due to the disruption of normal coagulation by cardiopulmonary bypass, the use of post-operative AC, or acquired von Willebrand disease (thought to be a result of increased shear stress) [51,52,53]. Bleeding occurs in 30–60% of patients after LVAD implantation, in the form of wound/drain bleeding in the early postoperative period or GI bleeding and epistaxis in the late postoperative period [54,55]. LVAD patients must be on therapeutic AC to reduce the risk of thrombosis, another common device-related complication. Clots can develop, causing pump thrombosis, embolism, or stroke, which can prove fatal. Najjar et al. showed a pump thrombosis rate of 8.1% in their cohort, consistent with rates found in other studies, namely Kirklin et al. [56,57]. Hemolysis can also occur post-operatively due to pump structure, cannula position, or the development of heparin-induced thrombocytopenia [58]. LVAD placement unloads LV pressure and allows the interventricular septum to shift back toward the left. It also provides for increased cardiac output, increasing the venous return to the RV. This increased RV filling concomitant with pulmonary HTN (a common sequela of severe HF) can compound RV filling pressures and lead to RV failure, necessitating the use of milrinone or even ECMO [59,60,61].

Infection is another common LVAD complication that is subdivided into LVAD-specific (driveline, pump site, and pump pocket), LVAD-related (bacteremia, mediastinitis, endocarditis), and non-LVAD related (urinary tract infection, pneumonia) causes [62]. The most common LVAD-specific infection is of the driveline, and the most common pathogen is *Staph aureus*, followed by *Pseudomonas* [62]. Infection treatment includes antibiotics, revision of driveline placement, surgical debridement, or explantation for severe infection [63]. Both hemorrhagic and ischemic strokes can occur after LVAD placement. Right-sided strokes are more common and are suggestive of an embolic cause [64,65]. Lastly, as previously mentioned, LVAD placement and recovery are associated with ventricular arrhythmia [48]. Cannula placement, suction, and scar tissue development can each alter cardiac electrical conduction and are associated with arrhythmia, which can be managed with anti-arrhythmic medication, changes in LVAD settings (i.e., decreased device flow), or ablation if still unresolved [66].

## 4. LVAD Outcomes and Relationship with DM

### 4.1. Outcomes after LVAD Implantation

Changes to the United States’ heart allocation system, as well as improvements in LVAD outcomes, have led to an increase in the number of LVADs being implanted for a DT rather than a BTT (DT indications increased from 44.6% in 2012 to 81.1% in 2021) [47]. In early trials, the implantation of first-generation, pulsatile LVADs as a DT in patients with severe HF who were ineligible for cardiac transplantation resulted in reductions in all-cause mortality by 48% and 50% in the REMATCH and INTrEPID studies, respectively [67,68]. Survival outcomes have continued to improve with the development of newer LVAD technologies. The probability of surviving to 5 years in patients implanted between 2017 and 2021 was 30.8% compared to 18.6% in patients between 2012 and 2016 [47]. Rates of neurologic dysfunction, one of the most feared complications of LVAD implantation, have decreased as well. In the REMATCH trial, neurologic events were 4.35 times higher in patients receiving LVAD implantation, while in INTrEPID, 62% of LVAD patients had a stroke or transient ischemic attack (TIA) within the study timeframe, compared with 11% in the non-LVAD group. In a more recent comparison, the rates of neurologic dysfunction significantly decreased in a 2017–2021 cohort compared to a 2012–2016 group (one-year freedom from a first stroke at 88.7% vs. 86.1%) [47]. Additionally, pump thrombosis, device malfunction, and gastrointestinal bleeding were significantly reduced in the later 2017–2021 group [47]. The most common adverse effects associated with CF-LVAD implantation among these two recent groups were major infection and bleeding, and rates remained similar between the two groups [47]. More recent studies comparing fully magnetic devices, such as the HeartMate III™, and other LVADs have demonstrated higher survival rates and lower incidences of gastrointestinal bleeding, stroke, and device malfunction/pump thrombosis among the fully magnetic LVADs [45]. Still, infection rates have remained similar [45], demonstrating an important area of improvement for future LVAD systems.

Kidney disease is commonly comorbid with HF in patients and can either be a result of independent/pre-existing kidney disease or a sequela of cardiorenal syndrome (CRS). Kidney disease can cause uremia, which, in patients undergoing cardiac surgery, can interfere with platelet and immune cell function. This can promote increased bleeding and disruption of wound healing, respectively [69]. Pre-operative kidney disease has been shown in 25–40% of LVAD recipients and is associated with increased all-cause mortality post LVAD implantation [9,70,71]. The prognosis for patients with end-stage renal disease (ESRD) is even worse. An 11-year retrospective-cohort study demonstrated an 81.9% mortality rate (median survival = 16 days) in patients with ESRD versus 36.4% in patients without ESRD (median survival = 2125 days) following LVAD placement [72]. Changes in kidney perfusion can occur from intraoperative cardiopulmonary bypass, aortic cross-clamping, vasopressors, and blood loss. The result can be either ischemic or reperfusion damage, which can further decrease kidney function [73]. Analysis of 15,754 patients from the INTERMACS database from 2006 to 2014 found that 12.3% of patients who received MCS developed acute kidney injury to the point of needing hemodialysis (HD) [74]. In a single-center retrospective cohort study, Asleh et al. found that 15% of patients required renal replacement therapy (RRT) after LVAD implantation (33% recovered kidney function, 33% required outpatient HD, and 33% died while an inpatient), and a longer time on cardiopulmonary bypass was an independent predictor of RRT requirement post LVAD placement [75]. Alternatively, there is evidence that the improved cardiac output after LVAD placement causes increased kidney perfusion and decreased central vascular congestion, leading to the recovery of kidney function. The literature has demonstrated that up to 50–60% of LVAD patients experience some level of renal recovery post-operatively, with the greatest increase occurring in the first three months after implantation [76]. Kilic et al. defined recovery as post-operative GFR > 60 mL/min/1.73 m^2^ and found that renal recovery was maintained at one year after implantation [77]. Unfortunately, this recovery may only be temporary, as there is evidence that patients experience worsening kidney function beyond one year [78].

### 4.2. LVADs and Glycemic Control

Several studies have shown that following LVAD implantation, A1c levels are statistically decreased at follow-up visits in the years after device placement [29,79,80,81,82,83,84]. For example, in a retrospective cohort study of 83 patients with T2DM who underwent LVAD placement, A1c levels decreased from 7.46% preoperatively to 6.21% postoperatively (*p* < 0.001), with the median postoperative A1c level taken at 4.8 months after LVAD placement [82]. Furthermore, A1c levels remained significantly lower than preoperative levels up to 24 months postoperatively [82]. This was likely not due to increased insulin adherence, as the total daily insulin requirement and non-insulin diabetic medication usage decreased during this time frame as well [82]. This general trend was mirrored in other studies. In a retrospective study in which 244 LVAD recipients were reviewed, A1c levels were significantly reduced 6 months post LVAD implantation (7.2% vs. 6.1%, *p* < 0.0001), with significant decreases in oral antidiabetic medication dosages [81]. In another retrospective study of 202 patients who underwent LVAD implantation (50 patients with T2DM), fasting blood glucose improved from 136 to 108 mg/dL post LVAD implantation (*p* < 0.001), and decreases in daily insulin dosages were noted [84].

While tighter glucose control in the hospital due to stricter dietary and insulin therapy could explain the improved A1c levels obtained at discharge or earlier follow-up visits, it would be unlikely to account for the improvements seen up to two years post LVAD implantation. On the other hand, the need for LVAD implantation as a treatment of HF may have been a motivating factor for patients to pursue steps to control other comorbidities, such as DM, through personal means such as diet, exercise, and closer adherence to prescribed regimens. While there has been no report to our knowledge on healthy lifestyle adherence following LVAD implantation, there have been similar studies following cardiovascular events. A study of medication adherence following myocardial infarction showed a significant decrease in patient adherence over a timeframe of one year, from a level of adherence of 65.0% in the first quarter to 50.7% in the final quarter [85]. Additionally, prior diagnoses of CAD and myocardial infarction, prior interventions such as percutaneous coronary intervention and coronary artery bypass grafting, and older age were all associated with worse levels of medication adherence [85]. As the LVAD implantation demographic is older and involves established diagnoses of HF, long-term adherence to healthy regimens may be similarly low. However, further studies on adherence to factors such as dieting, exercising, and medication compliance in patients who received an LVAD could be more informative.

Alternatively, LVADs have been shown to increase exercise capacity and improve quality of life [86]. Exercise is associated with improved glucose levels through direct muscle uptake and increased muscle sensitivity to insulin [87]. However, multiple studies have reported an improved A1c without associated improvements in body mass index (BMI) [29,80,81,82,83], making benefits attributable to diet/exercise less likely. There remains the possibility that these results could partially be explained by increased muscle mass following LVAD implantation, accounting for the similar BMI despite the loss of fat, as a high muscle to fat ratio has been associated with increased insulin sensitivity [88]. BMI alone may be a limited surrogate for an intervention that improves exercise capacity, as it will be unable to discern between changes in fat and muscle mass. Further studies elucidating the proportion of fat to muscle in patients post LVAD implantation might shed more light on whether LVADs lead to a favorable change in tissue despite minimal changes in BMI.

Improved cardiac output and organ perfusion, leading to decreased catecholamine and cortisol levels, have also been proposed as mechanisms of better glycemic control, and markers of renal function, such as creatinine, have demonstrated evidence of increased perfusion following LVAD implantation [29,79,80,81,83]. Increased perfusion of the pancreas following LVAD implantation may lead to decreased insulin resistance, although studies evaluating pancreatic perfusion following LVAD implantation are limited. Additionally, while A1c levels often show a dramatic decrease within a few months of LVAD placement, studies with longer-term follow-up have demonstrated slight increases in A1c (albeit still significantly reduced compared to pre-operative A1c levels). In Yen et al.’s study, A1c levels were 7.46% pre-LVAD, 6.19% 1–6 months post implantation, 6.42% at 6–12 months, and 6.62% at 12–24 months [82]. In Guglin et al.’s study, A1c levels were 7.56% pre-LVAD implantation, 5.47% 0–3 months post therapy, 6.1% at 3–6 months, 6.29% at 6–9 months, and they decreased to 5.26% at 9–12 months after LVAD implantation [80]. Yen et al. postulated that these results may be attributable to factors including the progression of DM despite restored cardiac output, the progression of underlying HF leading to worsening organ perfusion, the observed increased BMI seen in the study offsetting the LVAD’s benefits, or possibly decreased adherence to diet, exercise, and medication regimens with increased time after the implantation [82]. Whether the improvements provided by LVADs on hemoglobin A1c persist for several years following implantation remains to be seen.

### 4.3. LVADs, Adverse Events, and Mortality in DM

While the impact of LVAD implantation on A1c level has been consistent across multiple studies, the effect of LVADs on mortality in diabetic patients has been more varied. The summarized findings of several studies investigating this principal question can be seen in Table 1. While some studies have demonstrated an increased risk of mortality following continuous LVAD implantation [27,89,90,91,92,93,94], other studies have shown no difference between diabetics and non-diabetics [95,96,97]. Similarly, adverse effects, including infection, device thrombosis, and neurologic events such as TIA, stroke, or intracerebral hemorrhage, have varied among different studies. Several studies have found no significant difference between any of these adverse events in diabetics, even within studies where a difference in mortality was observed [90,91,95,96,98]. The effect on pre-LVAD glycemic control also does not appear to significantly influence mortality, as several studies have reported no association between better A1c control (usually defined as A1c < 7%) and subsequent mortality outcomes among diabetic groups [90,95].

In a single-center study of 341 patients who received an LVAD between 2007 and 2017, 78.8% of patients received HeartMate III™ and 15% received HeartWare^®^ [90]. In this study, the all-cause mortality rate (hazard ratio (HR) 1.73; 95% CI: 1.18–2.53; *p* = 0.005) and the device infection rate (HR 2.1; 95% CI: 1.35–3.18; *p* = 0.001) were both significantly higher among diabetics. Additionally, while the rates of pump thrombosis and stroke/TIA were not significantly different, the composite endpoint of all thromboembolic event points was greater in the diabetic group (unadjusted HR 1.61; 95% CI: 1.04–2.45; *p* = 0.03) [90]. Usoh et al. found that in a study of 191 patients who received HeartMate II™ between 2008 and 2014, DM was associated with a higher cumulative probability of death at three years (42% vs. 21%, *p* = 0.013), with no significant differences in overall rates of infection or neurologic dysfunction [91]. Kogan et al. studied the effects of DM in patients who received either HeartMate III™ (65%) or HeartMate II™ (27%) and found that while mortality was higher among diabetics 30 days (16.1% vs. 9.8%, *p* = 0.312), 1 year (24.2% vs. 17.3%, *p* = 0.399) and 3 years (30.6% vs. 21.9%, *p* = 0.127) following LVAD implantation, it did not reach significance until 5 years (38.7% vs. 24.4%, *p* = 0.038) [94]. The rate of major infections was higher in the diabetic group, but the rates of neurologic dysfunction, renal dysfunction, major bleeding, and device malfunction were similar [94]. Two separate analyses of the INTERMACS registry, allowing for the evaluation of a larger number of LVAD patients (>1000), found that the absence of DM was associated with increased odds of survival at 1–3 years [89,93].

On the other hand, several trials have found no significant difference in mortality. In a retrospective, single-center study of 300 patients with an LVAD placed between 2006 and 2013, DM was not associated with all-cause mortality (HR 0.883; 95% CI: 0.571–1.366; *p* = 0.5768) or with adverse endpoints of stroke/TIA, intracerebral hemorrhage, pump thrombosis, or infections [95]. In another study of 244 patients who received HeartMate II™ and 44 who received HeartWare^®^, survival was not significantly different at mean follow-up durations of approximately 1100 days, with an HR of 0.99 (95% CI, 0.65–1.6; *p* = 0.97) [96]. Additionally, there was no difference in the rates of GI bleeding, intracerebral hemorrhage, stroke, LVAD infection, or pump exchange, and hemolysis was the only adverse effect studied that was significantly more prevalent in the diabetic group [96].

This variation in all-cause mortality persisted among meta-analyses. A meta-analysis of six studies including 1543 patients (64.7% HeartMate III™, 27.7% pulsatile LVAD, and 7.5% HeartWare^®^) found that the overall mortality rate at three years was higher among diabetics and diabetics had higher rates of strokes. Still, other adverse effects, including infections, pump thrombosis, renal failure, and bleeding, were similar between diabetics and non-diabetics [92]. A meta-analysis of 1120 patients across four studies receiving HeartMate II™ or HeartWare^®^ found that DM was not associated with a significantly higher HR for overall mortality, and no significant difference was found between the two groups regarding infection, intracranial hemorrhage, or pump thrombosis [97]. In an analysis of 4978 patients who underwent LVAD implantation (HeartMate II™ or HeartWare^®^, from 2000 to 2015) as part of a BTT, all-cause mortality was not higher following LVAD implantation [98]. However, in the 3058 patients who underwent subsequent cardiac transplantation, having comorbid DM was associated with a significantly lower survival rate compared to those without DM at 1-, 3-, and 9-year follow-ups (87% vs. 91%, 79% vs. 84%, 60% vs. 64%, respectively, *p* (log-rank) = 0.001) [98].

In a single-center retrospective study of 212 patients who underwent LVAD placement, those with post-implantation infection were more likely to be diabetic (42.4% vs. 24.8%, *p* < 0.001) [99]. The preoperative diagnosis of DM was the only risk-adjusted independent predictor of infection in this study. There are also links found between infection and cerebrovascular events (CVAs). In a multicenter retrospective analysis of 3282 patients following LVAD implantation, 38.5% of these patients suffered from an LVAD-related or LVAD-specific infection, and 14.8% of patients experienced a hemorrhagic or ischemic stroke within a 3-year follow-up period [100]. There was a significant increase in the hazard ratio for CVA at 1.90 (95% CI: 1.55–2.33, *p* < 0.001) during infection in LVAD patients [100]. In sepsis, the blood becomes hypercoagulable with increased platelet activation and aggregation in the setting of inflammation [101]. Combined with the sheer stress of continuous flow in LVADs resulting in acquired von Willebrand syndrome, as mentioned earlier, the risk of ischemic and hemorrhagic CVAs can theoretically be increased [51,53].

## 5. Future Directions

As demonstrated, the literature is abundant regarding the impact of DM on short- and long-term outcomes (i.e., all-cause mortality and significant adverse events) after LVAD implantation, with no clear consensus. Single-center prospective trials can provide the most specific patient demographic and outcome data but suffer from small sample sizes and loss to follow-up. Meanwhile, large-scale meta-analyses or national database projects are unable to provide specific data on each patient, such as the cause of death (i.e., LVAD-specific or unrelated), DM type (T1DM vs. T2DM), or level of glycemic control (A1c). There remains the need for a large-scale meta-analysis that investigates the impact of DM on short- and long-term outcomes after LVAD implantation with sufficient power to detect significance and details regarding patient outcomes that can inform clinical decision-making.

Additionally, many of the studies examined in this review investigated differences in outcomes among diabetic patients from the mid-2000s to the mid-2010s. Because of this, the LVADs implanted were older devices, predominantly HeartMate II™ or HeartWare^®^. However, with the development of newer technologies, overall mortality and rates of many adverse effects have continued to improve in patients receiving LVADs [45]. Kogan et al. examined a patient population who predominantly received the newer HeartMate III™ (65.9% of the diabetic group, 65.2% of the non-diabetic group) and found that mortality differences were significantly higher in diabetics at 5 years [94]. As HeartMate III™ is the only system currently implanted in the United States, further investigations focusing on the HeartMate III™ are important to discern whether significant adverse events and mortality outcomes differ between diabetics and non-diabetics.

Regarding the renal impact of LVADs, while there are significant data on early renal recovery in patients with kidney disease after LVAD implantation, recent studies suggest that this may be a false positive. Most studies use estimated GFR to assess kidney function, calculated from serum creatinine [76]. Many LVAD patients are chronically ill and experience muscle wasting, which may overestimate GFR. Furthermore, LVAD patients can experience increased muscle wasting post-operatively due to the impact of surgery and immobility, possibly confounding post-operative renal recovery data [102]. Cystatin C has been suggested as a superior biomarker, but it too has been associated with muscle mass in HF patients [103]. As previously discussed, DM has an important impact on the development and progression of CKD. The inextricable link between DM, cardiac, and renal disease has been discussed here, as illustrated by the changes in A1c and renal function after LVAD placement. While the accuracy and sustainability of the apparent improvement in A1c and renal function are still a subject of investigation, it is possible that a further understanding of the impact of LVAD implantation on glycemic control and, in turn, kidney function in diabetic LVAD patients may provide insights into their long-term outcomes. While many studies examining diabetic patients have focused on important diabetic complications such as CVA, pump thrombosis, and infections, studies assessing renal function are more limited. Those studies that have included renal dysfunction often show no differences with diabetics. All of this indicates a need to identify a more accurate biomarker of kidney function in LVAD patients, along with larger-scale studies investigating the bidirectional connections between LVADs, glycemic control, and renal outcomes.

Finally, many of the examined studies focus on the effect of LVAD implantation on overall survival and how DM could lead to adverse effects within the LVAD patient population, such as through CVA, infections, pump thrombosis, and infections. However, studies examining how LVADs affect complications of DM itself are more limited. While the examined studies overwhelmingly show that LVADs lead to a decrease in A1c following implantation, future studies could further examine whether this translates to a reduction in diabetic-related complications (i.e., peripheral neuropathy, retinopathy, etc.) and/or an improvement in patients’ quality of life.

## 6. Conclusions

The primary focus of this narrative review was to investigate the role of DM in LVAD outcomes, both in the short term and the long term. While there seems to be a correlation (although not necessarily a causation) regarding decreased A1c following LVAD implantation, the impact that this has on clinical diabetic complications and the sustainability of this impact remain undetermined. Most importantly, the literature regarding major adverse outcomes and mortality in DM patients after LVAD placement remains without consensus. Given the significant differences found in several reviewed studies, a larger sample size or longer follow-up time may be required to reveal significance. It may also be that LVAD complications and mortality are not one-size-fits-all problems. LVAD implantation and maintenance comprise a complex process, and patients often have numerous comorbidities and require comprehensive health maintenance afterward. Analyzing the impact of individual patient factors such as DM type, the severity of comorbidities, glycemic control, medication adherence, and the specific cause of death are all possible directions for further research and may elucidate the true impact of DM on LVAD outcomes.

## Figures and Tables

**Table 1:** Outcomes in DM Patients After LVAD Implantation.

Authors, Ref.	Study Design	Duration	Population	LVAD Model	Key Findings	*p*-Value
Butler et al., 2005 [27]	Retrospective cohort	1996–2003	222 Patients	Novacor	Patients with DM had higher mortality compared to nondiabetic patients at 30, 180, and 365 days after implantation	*p* = 0.02
Arnold et al., 2016 [89]	Retrospective cohort	2012–2013	1638 Patients	Not specified	Patients with severe DM had a significantly higher rate of poor outcomes (limiting HF symptoms, poor quality of life, or death) compared to non-DM patients one year after implantation	*p* = 0.038
Vest et al., 2016 [95]	Single-center retrospective cohort	2006–2013	300 Patients	HeartMate II™, HeartWare^®^	DM was not associated with significantly higher rates of all-cause mortality or major adverse events (stroke/TIA, ICH, pump thrombosis, infection)	*p* = 0.5768
Asleh et al., 2017 [90]	Single-center retrospective cohort	2007–2017	341 Patients	HeartMate II™, HeartMate III™, HeartWare^®^, Jarvik 2000, DH DuraHeart	All-cause mortality and device infections were significantly more common in diabetic vs. non-diabetic patients	*p* = 0.03
Mohamedali et al., 2017 [96]	Single-center retrospective cohort	2006–2013	288 Patients	HeartMate II™, HeartWare^®^	Other than hemolysis, there was no significant difference in the rate of adverse events or mortality in patients with versus without DM after LVAD placement	*p* = 0.71
Usoh et al., 2018 [91]	Single-center retrospective cohort	2008–2014	191 Patients	HeartMate II™	DM patients had a higher cumulative probability of death at three years compared to non-diabetic patients. No significant difference was found in rates of infection or neurologic dysfunction	*p* = 0.013
Blumer et al., 2018 [92]	Systematic Review	All available data through 2017	1543 Patients	HeartMate II™, Pulsatile LVAD, HeartWare^®^	The rates of stroke and mortality were significantly higher among DM patients compared to non-diabetics.	*p* = 0.01
Al-Kindi et al., 2019 [98]	Retrospective cohort	2000–2015	4978 Patients	HeartMate II™, HeartWare^®^	There was no significant difference in all-cause mortality between patients with and without DM.	*p* = 0.30
Xia et al., 2019 [93]	Retrospective cohort	2012–2013	1116 Patients	Not specified	DM was associated with significantly increased odds of death 3 years after implantation	*p* ≤ 0.01
Zhou et al., 2020 [97]	Meta-analysis	4 studies (2016–2018)	1120 Patients	HeartMate II™, HeartWare^®^	No significant difference in overall mortality or adverse events (infection, ICH, pump thrombosis) between patients with and without DM	*p* = 0.18
Kogan et al., 2022 [94]	Single-center retrospective cohort	2006–2020	154 Patients	HeartMate II™, HeartMate III™, HeartWare^®^	Patients with DM had a significantly higher mortality rate compared to non-diabetics 5 years after implantation. Infection rate was also significantly higher in patients with DM	*p* = 0.038
